# Smart Sensor for Real-Time Quantification of Common Symptoms Present in Unhealthy Plants

**DOI:** 10.3390/s120100784

**Published:** 2012-01-11

**Authors:** Luis M. Contreras-Medina, Roque A. Osornio-Rios, Irineo Torres-Pacheco, Rene de J. Romero-Troncoso, Ramon G. Guevara-González, Jesus R. Millan-Almaraz

**Affiliations:** 1 HSPdigital-CA Mecatrónica, Facultad de Ingeniería, Universidad Autónoma de Querétaro, Campus San Juan del Rio, Rio Moctezuma 249, 76807 San Juan del Rio, Qro., México; E-Mails: mcontreras@hspdigital.org (L.M.C.-M.); troncoso@hspdigital.org (R.J.R.-T.); 2 Ingeniería de Biosistemas CA, División de Estudios de Posgrado, Facultad de Ingeniería, Universidad Autónoma de Querétaro, Cerro de las Campanas S/N, 76010 Querétaro, Qro., México; E-Mails: irineo.torres@uaq.mx (I.T.-P.); ramon.guevara@uaq.mx (R.G.G.-G.); 3 Facultad de Ciencias Físico-Matemáticas, Universidad Autónoma de Sinaloa, Av. De las Américas y Blvd., Universitarios, Cd. Universitaria, 80000 Culiacán, Sin., México; E-Mail: jrmillan@uas.edu.mx

**Keywords:** smart sensors, symptoms in plants, computer vision, image processing, plant diseases, FPGA

## Abstract

Plant responses to physiological function disorders are called symptoms and they are caused principally by pathogens and nutritional deficiencies. Plant symptoms are commonly used as indicators of the health and nutrition status of plants. Nowadays, the most popular method to quantify plant symptoms is based on visual estimations, consisting on evaluations that raters give based on their observation of plant symptoms; however, this method is inaccurate and imprecise because of its obvious subjectivity. Computational Vision has been employed in plant symptom quantification because of its accuracy and precision. Nevertheless, the systems developed so far lack *in-situ*, real-time and multi-symptom analysis. There exist methods to obtain information about the health and nutritional status of plants based on reflectance and chlorophyll fluorescence, but they use expensive equipment and are frequently destructive. Therefore, systems able of quantifying plant symptoms overcoming the aforementioned disadvantages that can serve as indicators of health and nutrition in plants are desirable. This paper reports an FPGA-based smart sensor able to perform non-destructive, real-time and *in-situ* analysis of leaf images to quantify multiple symptoms presented by diseased and malnourished plants; this system can serve as indicator of the health and nutrition in plants. The effectiveness of the proposed smart-sensor was successfully tested by analyzing diseased and malnourished plants.

## 1. Introduction

In [1,2], symptoms in plants are defined as responses to alterations of their physiological functions and metabolic disorders that are caused principally by infections or nutritional deficiencies. The symptoms can be frequently visualized in leaves, stem and roots, so that precise assessments of plant symptoms can be used as indirect indicators for monitoring disease and malnutrition in plants, estimating yield loss and breeding for resistance against plant diseases [3].

In plant pathology, a pathogen is defined as the agent that causes the disease and a diseased plant is described as visible and invisible responses (symptoms) of cells and tissues to pathogens or environmental factors that can produce alterations in the morphology, physiological functions and integrity of the plant that may cause partial or complete damage [1]. In [1,4] it is mentioned that plant diseases cause up to 14.1% of the worldwide agricultural production losses; this fact results in environmental, economical and health problems. Therefore, disease assessment is critical to various aspect of the study of plant pathogens [5]. Good quality disease assessment data is needed to make appropriate decisions in disease management, to compare treatments, monitor plant diseases and gauge cultivar resistance in plant breeding [5,6].

The study of how the plants obtain and use mineral nutrients is called mineral nutrition; this is central research in modern agriculture because high agricultural yields strongly depend on fertilization with mineral nutrients and before saturation the yields of most crops increase proportionally with the amount of fertilizer that the plants absorb; therefore, mineral nutrition research is necessary to meet the incremental worldwide demand for food [2]. Nutrient deficiency symptoms are the expressions of metabolic disorders resulting from insufficient supply of essential elements; in consequence, precise assessments of these symptoms can be used as reliable nutritional deficiency indicators in order to compare treatments and for fertilizer management.

Nowadays researchers have developed techniques to measure the symptoms presented in plants, and the visual estimation (VE), which implies the use of trained personnel that evaluate the health and nutrition in plants based on visual symptoms, is the most popular [7]. According to Bock *et al*. [7] VE can be used as disease assessment method, as can be seen in [8,9], where individual plants were rated for symptoms using a four-level and nine-level based disease severity index, respectively, in order to know the resistance of common bean to bean dwarf-mosaic virus and resistance of pepper to geminiviruses. Nevertheless, VE is not able to give quantitative evaluations because it introduces subjectivity, inaccuracy and imprecision [3,5,10]. To overcome these drawbacks, computer vision (CV), which is based on Image Analysis (IA), can be used as a disease assessment method [7]; CV is based on plant symptom quantification and compared to VE, offers higher precision, accuracy and reproducibility assessments, however, according to Bock *et al*. [5], it is incapable of distinguishing particular symptoms of a diseased plant when the plant is affected by various diseases. Martin and Rybicki [11] realized a comparative between commercial CV-based system and custom CV-based system evaluations of chlorotic lesion areas occurring on leaves of *Zea mays* provoked by maize streak virus (MSV). In [12] a CV-based system was used to classify visual symptoms caused by angular bacteria, *Ascochyta blight*, and green stink bug through analysis of coloured images and by using techniques such as co-occurrence matrix, fractal dimension, lacunarity and a support vector machine (SVM). Camargo and Smith [13] proposed a CV-based methodology to identify disease symptoms of photographed leaves based on H, I3a and I3b colour transformation and local maximums localized in the histogram. Wijekoon *et al*. [14] realize a quantification of fungal infections on leaves of *N. benthamiana* infected with anthracnose pathogen, using diagrams of different diseases proposed by James [15], the images were captured using a flat scanner and camera to later being analyzed in the laboratory using CV. Al-Hiary *et al*. [16], proposed an CV-based algorithm to classify diseased leaves affected by different pathogens; the algorithm is based on the computation of texture statistics and neural networks to accomplish the classification; the algorithm detects and classifies the examined plant diseases with a precision between of 83% and 94%. Even thought the aforementioned CV-based systems give precise and accurate assessments, they are time-consuming, not-easy to use and frequently analyze just one specific plant symptom.

Optically-based methods commonly used to detect symptoms provoked by viral, bacterial and fungal disease are thermography, hyperspectral reflectance, chlorophyll fluorescence-based techniques, nuclear magnetic resonance (NMR) spectroscopy and X-ray imaging [17]. According to Chaerle *et al*. [18], thermography allows one to detect symptoms in plants before they can be detected by an evaluator, so that, local temperature changes due to plant defence mechanism against disease can be monitored employing this method [17]. The hyperspectral reflectance technique allows obtaining a signature at varying wavelengths in the visible, near-infrared, and shortwave-infrared range of the electromagnetic spectrum. Delaieaux *et al*. [19] used hyperspectral reflectance to detect apple scab caused by *Venturia inaequalis*; to accomplish that, it was necessary to select the wavelengths best suited for classifying infected leaves from those of the healthy leaves. Chlorophyll fluorescence-based techniques involve fluorescence spectroscopy and fluorescence imaging; both can be used to monitor nutrient deficiencies, environmental-condition-based stress levels and diseases in plants [17,18]. Belasque *et al*. [20] employed fluorescence spectroscopy to detect stress caused by citrus canker and mechanical injury and Bravo *et al*. [21] utilized fluorescence imaging for detecting yellow rust in winter wheat. Similarly, nuclear magnetic resonance and X-ray imaging techniques can be used for detecting infections, different types of stress and health conditions in plants [17,22]. Goodman *et al*. [23] used NMR microscopic imaging for identification of the fungal pathogen *Botrytis cinerea* in red raspberry and Navakar *et al*. [24] applied X-ray imaging to identify fungal infections in wheat. Those optical-based techniques present the disadvantages of needing expensive equipment, trained personnel and time-consuming.

Volatile organic compounds (VOC) released by plants are influenced by physico-chemical factors (humidity, temperature, light, soil condition, growth and developmental stage) that affect the physiological condition of the plant, thereby influencing the VOC profile [17]. Abiotic and biotic stresses can provoke changes in the VOC profiles of the plants; this can be utilized as an indirect indicator for plant disease assessment. The two common methods for assessing the profile of volatile metabolites released by plants are gas chromatography (GC)-based and electronic-nose-based system techniques. Laothawornkitkul *et al.* [25] evaluated the potential of plant volatile signatures for pest and disease monitoring in cucumber, pepper and tomato plants. Liu *et al*. [26] inoculated potato tubers with *Phytophthora infestans, Pythium ultimum*, or *Botrytis cinerea* and analyzed the resulting VOC profiles using GC. The VOC-based systems developed so far for monitoring plant disease are not able to achieve *in-situ* and time-consuming monitoring and also present the disadvantage of the natural variation in the VOC profile within plant species [17] and expensiveness.

The most popular molecular techniques for plant disease assessment are Polymerase Chain Reaction (PCR) and Enzyme-Linked ImmunoSorbent Assay (ELISA); they have the robustness and capacity to provide plant disease assessments by quantifying the amount of virus, bacteria and fungi in plant tissue as can be seen in [27], where the PCR technique was used to quantify the degree of colonization of English oak (*Quercus robur L.*) leaves by the bacteria *Erwinia*; in the same way, Gutiérrez-Aguirre *et al*. [28] use PCR and ELISA techniques to detect cucumber mosaic virus in tomato plants. Although the molecular techniques offer high precision and accuracy, they present the disadvantage of being destructive, time-consuming and labor-intensive methods; also, they require an elaborate procedure to obtain reliable and accurate results [17].

The aforementioned techniques and works are used for monitoring different kinds of plant symptoms and they can be used as plant disease assessment; in the case of the molecular methods, these assessments are obtained by quantifying directly the amount of pathogens. CV is based on IA and it is a technique that tries to solve the drawbacks of the aforementioned methods; however, greater automation is needed to make this practical [7]. Bock *et al*. [5] mention that where a large number of samples need to be assessed, automation is particularly advantageous to conserve time while ensuring accuracy and precision of measurement. Some commercial and custom image analysis have automated functions; yet, these systems have not been widely applied in measuring plant disease, perhaps due to their complexity and/or expense [5,11,29]. Therefore, a smart-sensors able to accomplish precise and accurate symptoms quantification that could serve to assess diseases and malnutrition in plants and incorporate characteristics such as: low-cost, real-time, non-destructive, and friendly using are desirable.

Frank [30] defined a smart sensor as a sensor that provides functions beyond those necessary for generating a correct interpretation of a sensed or controlled quantity, based on this; smart sensors must incorporate processing, communication and integration according to Rivera *et al*. [31]. Field Programmable Gate Arrays (FPGAs) are devices that have been gaining popularity principally because of their high-speed processing, high reconfigurability and System on a Chip (SoC) solutions [32]; these characteristics permit that the FPGAs be used in applications where high-performance computational capabilities are needed. In CV, the FPGA high-speed processing characteristic has been exploited to develop vision systems to classify crop products [33]. In biology Millan-Almaraz *et al.* [34] used an FPGA-based smart sensor to estimate the plant-transpiration dynamics based on five primary sensors that measure air and leaf temperature, air relative humidity, plant out relative humidity and ambient light.

Because of the aforementioned reasons, this work proposes a low-cost and FPGA-based smart sensor for the quantification of common symptoms presented in diseased and malnourished plants by using CV-based, real-time, non-destructive and *in-situ* analysis of plant-leaf images. The proposed system employs a camera (1/3-inch Megapixel CMOS Active-Pixel Digital Image Sensor MT9M011 manufactured by Micron) [35] as primary sensor and an Altera DE2 development kit containing an EP2C35F672C6N Cyclone II FPGA as processing element or Hardware Signal Processing (HSP) unit. The smart sensor uses several novel colour-based methodologies that make use of morphological image processing and different colour components; these methodologies permit one to choose the appropriate colour component according to the symptom under analysis, always trying to diminish the noise introduced by other symptoms (not under analysis at that moment) that are present in a sample. In this work, the studied symptoms are: chlorosis, leaf deformation, white spots, necrosis, and mosaics because they are common symptoms present in diseased and malnourished plants. The smart-sensor functionality was successfully tested by analyzing quantitatively the aforementioned symptoms present in diseased bean and pepper plants, and malnourished pumpkin plants; the system gives numerical values, by using CV, that objectively describe the degree of the symptom present in the plant leaves. Because the plant symptoms are responses to the alteration of plant physiological functions provoked principally by infections or nutritional deficiencies, the system could be employed, in future works, as a powerful tool to give quantitative descriptions of plant symptoms development; this quantitative descriptions could serve to assess disease and malnourishment in plants.

## 2. Symptoms in Plants

Symptoms in plants are commonly present in diseased and malnourished plants, and are caused principally by pathogens and mineral deficiencies. Five common symptoms in plants are: chlorosis, leaf deformation, white spots, necrosis, and mosaics. In this work, bean plants (*Phaseolus vulgaris*), pepper plants (*Capsicum annum*), and pumpkin plants (*Curcubita pepo*) were analyzed. Bean plants were infected with the common bean mosaic virus; pepper plants were infected by the bacteria *Xanthomonas campestris* and pumpkin plants were affected just by nutritional deficiencies.

### 2.1. Chlorosis

**Figure 1.**
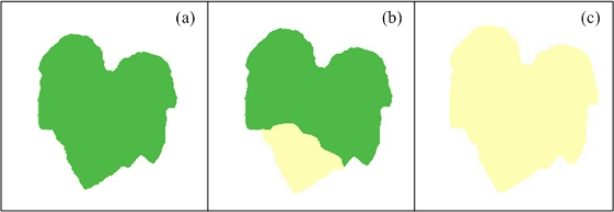
(**a**) Pumpkin leaf with healthy green colour. (**b**) Pumpkin leaf with localized chlorosis. (**c**) Pumpkin leaf with generalized chlorosis.

Chlorosis is defined as a yellowing of normally green leaf tissue that, in the case of plant diseases, could be due to chlorophyll destruction or failure in chlorophyll formation [1]. It could be caused by a variety of viral, bacterial and fungal pathogens. In the case of nutritional deficiencies, chlorosis is the most common symptom and it can be caused by several reasons, one of them is the lack of minerals that serve as constituent of many plant cells, coenzymes and vitamins and as essential components in the metabolism; these minerals are nitrogen and sulphur; other reason is due to the deficiencies of minerals such as potassium, calcium, and magnesium present within the plants as cations and ions playing an important role in the photosynthetic process [2]. Chlorosis symptoms can be localized or generalized, meaning that chlorosis can affect only certain leaf regions or the complete leaf. An ideal example of healthy leaf and localized and generalized chlorotic leaf can be seen in Figure 1, which shows schematized leaves with idealized symptoms. Figure 1(a) shows a healthy pumpkin leaf that has an idealized homogeneous green colour tissue; aside, Figure 1(b) shows a pumpkin leaf with idealized localized chlorosis; the leaf presents a section with homogeneous green colour tissue and the missing leaf section are covered by a yellowing colour. Figure 1(c) shows a pumpkin leaf with an idealized generalized chlorosis, the leaf presents a yellowing colour completely covering the leaf tissue.

### 2.2. Necrosis

**Figure 2.**
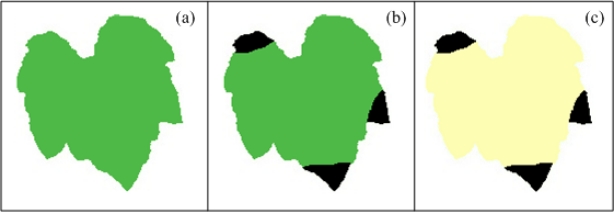
(**a**) Pumpkin leaf with homogeneous green colour. (**b**) Non-chlorotic pumpkin leaf with necrotic areas. (**c**) Chlorotic pumpkin leaf with necrotic areas.

Death in plant tissue in a localized area is called necrosis and commonly results in brown or black lesions often preceded by yellowing (chlorosis, or a breakdown in chlorophyll). This symptom can be provoked by plant pathogens or deficiencies of minerals such as boron, potassium, calcium, chlorine and sodium [2,36]. Figure 2 shows three schematized images that present how this symptom manifests over the plant leaves, Figure 2(a) presents a healthy pumpkin leaf with idealized green colour tissue, Figure 2(b) shows an unhealthy pumpkin leaf with idealized necrotic symptoms, and Figure 2(c) presents unhealthy pumpkin leaf with idealized necrotic and generalized chlorotic symptom. These three images show examples of how necrosis symptoms could appear in healthy and chlorotic leaves.

### 2.3. Leaf Deformation

**Figure 3.**
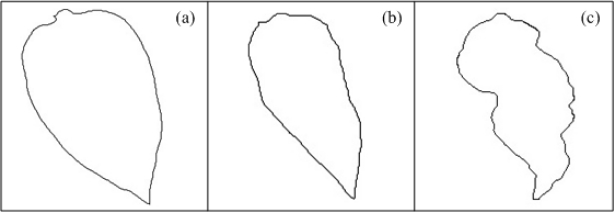
(**a**) Contour of healthy pepper leaf. (**b**) Contour of pepper leaf with low deformation. (**c**) Contour of pepper leaf with severe deformation.

Changes in the lamina of the host leaf result in areas that are twisted, deformed, or distorted; these deformations can be described as bubbles, rugosity or curls [36]; they can be caused by plant pathogens or mineral nutrition deficiencies, principally by absence of potassium [2]; these kinds of deformations imply a change in the normal shape of the leaf edge. Figure 3(a) presents the outline of a schematized healthy pepper leaf shape, Figure 3(b,c) shows the contour of schematized non-healthy leaves that present a deformation due to the presence of bubbles and roughness. The three figures show visually the presence of the leaf deformation symptom. It is clear how the healthy leaf presents a more symmetric shape than the other two leaves, these contours can be measured by obtaining leaf geometric characteristics.

### 2.4. White Spots

**Figure 4.**
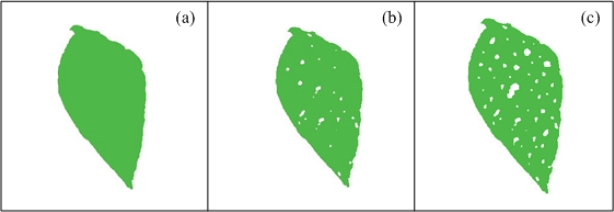
(**a**) Healthy bean leaf with green colour tissue. (**b**) Unhealthy bean leaf with low density of white-spot symptom. (**c**) Unhealthy bean leaf with high density of white-spot symptom.

White spots are provoked when plant pathogens or the deficiency of minerals involved in chlorophyll biosynthesis such as zinc, affect the photosynthesis and respiration functions; this leads to inability of the plant to respond to the pathogen [1,2]. Figure 4(a) presents a schematized healthy bean leaf with idealized green colour tissue and Figure 4(b,c) shows schematized unhealthy bean leaves with idealized white-spots symptom; as can be seen, the presence of white spots is clear in unhealthy leaf comparing with a healthy leaf. White spots can affect both chlorotic and non-chlorotic leaves.

### 2.5. Mosaics

**Figure 5.**
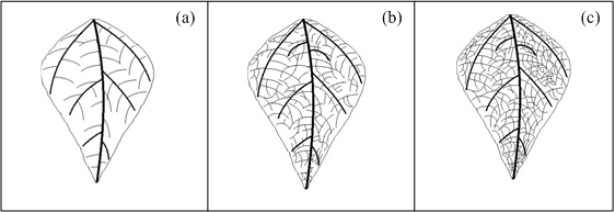
(**a**) Healthy bean leaf without mosaic symptom. (**b**) Unhealthy bean leaf with low-density mosaic symptom. (**c**) Unhealthy bean leaf with high-density mosaic symptom.

Mosaics are characterized by light-green, yellow, or white-areas intermingled with the normal green of the leaves or of lighter-coloured areas intermingled with areas of normal colour on flowers or fruits; this symptom is mainly provoked by viral pathogens [1]. Depending on the intensity or pattern of discolorations, mosaic-type symptoms may be variously described as mottling, steaks, ring patterns, line patterns, veinclearing, veinbanding, or chlorotic spotting [1]. Figure 5 shows three idealized veinclearing mosaic-type densities in leaves that are characterized by a clearing of the venation leaf. Figure 5(a) shows a venation diagram of schematized healthy bean leaf. Figure 5(b,c) presents a venation diagram of schematized unhealthy bean leaf with low-density and high-density mosaic symptom (veinclearing), respectively. It can be seen from the comparison between healthy and unhealthy leaves, how leaf venation becomes clearer in unhealthy leaves when it has the mosaic symptom.

## 3. Proposed Image Processing Algorithms

### 3.1. Chlorotic-Area Algorithm

**Figure 6.**
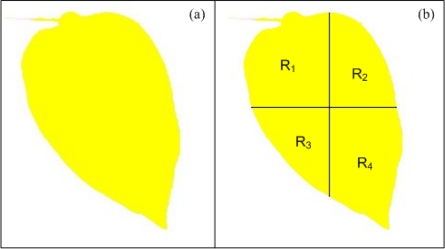
(**a**) Yellow component of healthy pepper leaf. (**b**) Sectioned healthy pepper leaf in four regions (R_1_, R_2_, R_3_, R_4_).

As aforementioned, a chlorotic area can be defined as a yellowing of normally green-leaf tissue; therefore, a good identification of chlorotic areas is done by using the yellow component of the image, resulting from a combination of red and green colormaps (Figure 6(a)). An algorithm based on colour analysis to quantify chlorotic level is proposed; it allows knowing if the chlorosis is generalized or localized. The algorithm can be divided in two stages, the first stage being in charge of calculating the yellow component of the leaf using the Equation (1); *Red* and *Green* correspond to red and green components of the original image, respectively. Then the leaf is divided into four sections as shown in Figure 6(b) using the centroid coordinates of the image *Cx* and *Cy*, depicted by Equation (2) [37] with *N* and *M* being the image row and column, respectively. The second stage is in charge of calculating the average yellow-level values of the four sections of the leaf by using Equation (3). *Yellow*(*i,j*) is the intensity level of the yellow component at the corresponding *(i,j)* index, *L_k_* is the number of pixels of the k section (where *k* = 1,2,3,4), and *i* and *j* are the line and column index, respectively. *R_k_* is the average yellow pixel value of *k* section; these values permit to calculate the modulus of a vector formed by these four average yellow level values *R_n_* as shown in Equation (4), and the modulus of the differential yellow level values vector *R_diff_* formed by subtracting the yellow level average value of each region from the other ones as shown in Equation (5); *R_n_* allow to quantitatively know how chlorotic is the leaf as a whole, whereas *R_diff_* permits to determine whether the chlorosis is localized or generalized:

(1) Yellow=0.5Red+0.5Green

(2) $$\mathrm{Cx}=\frac{\sum_{i=1}^{N}\sum_{j=1}^{M}i\cdot \mathrm{Yellow}(i,j)}{\sum_{i=1}^{N}\sum_{j=1}^{M}\mathrm{Yellow}(i,j)},\mathrm{Cy}=\frac{\sum_{i=1}^{N}\sum_{j=1}^{M}\;j\cdot \mathrm{Yellow}(i,j)}{\sum_{i=1}^{N}\sum_{j=1}^{M}\mathrm{Yellow}(i,j)}$$

(3) $$R_{K}=\left\{\frac{1}{L_{K}}\sum_{i=1}^{N}\sum_{j=1}^{M}\mathrm{Yellow}(i,j)|\mathrm{Yellow}(i,j)\varepsilon \;R_{k}\right\}$$

(4) $$R_{n}=\left\|\left[R_{1};R_{2};R_{3};R_{4}\right]\right\|$$

(5) $$R_{\mathrm{diff}}=\left\|\left[R_{1}-R_{2};R_{1}-R_{3};R_{1}-R_{4};R_{2}-R_{3};R_{2}-R_{4};R_{3}-R_{4}\right]\right\|$$

### 3.2. Necrotic-Area Algorithm

**Figure 7.**
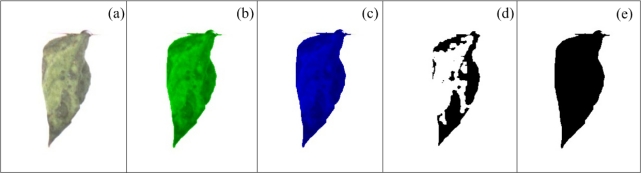
Bean leaves with necrosis. (**a**) Necrotic bean leaf in RGB format. (**b**) Green component of the necrotic bean leaf. (**c**) Blue component of the necrotic bean leaf. (**d**) Necrotic leaf area segmentation. (**e**) Total leaf area segmentation.

To quantify the necrotic area in leaves, a colour-based algorithm using the green and blue components is proposed. Figure 7(a) shows a bean leaf in RGB (Red, Green, Blue) format and its corresponding blue and green components are shown in Figure 7(b,c), respectively. Green component is used to isolate the necrotic area of the leaf (*A_np_*) from the total leaf and background because it offers a better contrast between necrotic and non-necrotic regions. The blue component is utilized to calculate the total leaf area *A_T_* because it is less sensitive to other symptoms such as chlorosis and it offers a better differentiation of leaf from the background. Before applying an image binarization, a median filter based on morphological opening *γ_B_* is accomplished; this operation is based on Equation (6) which in turn is based on morphological dilation *δ_B_* and erosion *ε_B_* (Equations (7) and (8)) [38]; this permits a better differentiation between necrotic and non-necrotic zones. Once quantified the necrotic and total leaf area in pixels as shown in Figure 7(d,e), necrotic area assessment *A_n_* is estimated by using the Equation (9). *A_n_* is the percentage of necrotic area covering the leaf relative to total leaf area:

(6) $$\gamma_{B}=A\circ B=(A\Theta B)⊕B$$

(7) $$\delta_{B}=A⊕B=\left\{z|\left[{\left(C\right)}_{z}\cap A\right]\subseteq A\right\}$$

(8) $$\varepsilon_{B}=A\Theta B=\left\{z|{\left(B\right)}_{z}\subseteq A\right\}$$

(9) $$A_{n}=\frac{A_{\mathrm{np}}}{A_{T}}*100\%$$

where *A* is the image and *B* is the structuring element. In Equation (7)*z* represents the set of displacements such that *C* (which is *B* reflected) and *A* overlap by at least one element and in Equation (8)*z* represents all points such that *B* translated in *A* is contained in *A*.

### 3.3. Leaf-Deformation Algorithm

**Figure 8.**
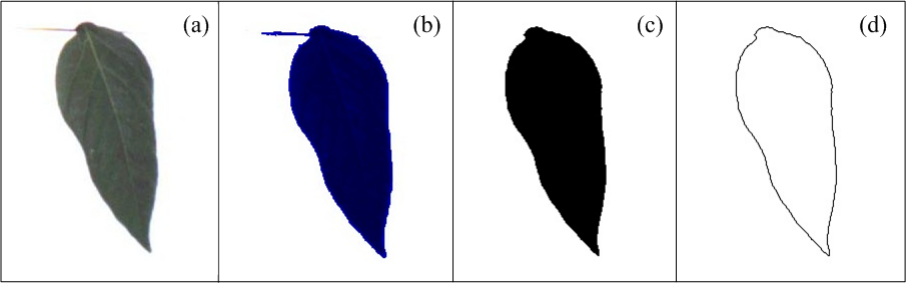
(**a**) Original pepper leaf. (**b**) Blue component of pepper leaf. (**c**) Leaf area segmentation (**d**) Leaf perimeter segmentation.

The methodology proposed for leaf-deformation assessment is a colour-based algorithm that uses, from the original image (Figure 8(a)), the blue component (Figure 8(b)) because it is less sensitive to other symptoms such as chlorosis that can provoke errors in the quantifications. Nowadays, there are several techniques to determine the geometric properties of an object; one of the most employed is the sphericity index [37]. This method gives a quantitative measurement about the sphericity of an object. To calculate this index it is necessary to obtain the area (*A*) and the perimeter (*p*) of the object (leaf) that, in the case of image processing, are calculated in pixels; Figure 8(c,d) shows the segmentation of the area and perimeter of the leaf respectively. When *A* and *p* are obtained, the sphericity index *I* is calculated using Equation (10) [37]; after this, *I* values of healthy and unhealthy (deformed) leaf are compared to quantitatively determine how the unhealthy leaf is deformed taking as reference the healthy leaf. In this work, *I* is also called deformation index because it is the variable that is measured to quantify the leaf deformation:

(10) $$I=\frac{p^{2}}{4\pi A}$$

### 3.4. White-Spots Algorithm

**Figure 9.**
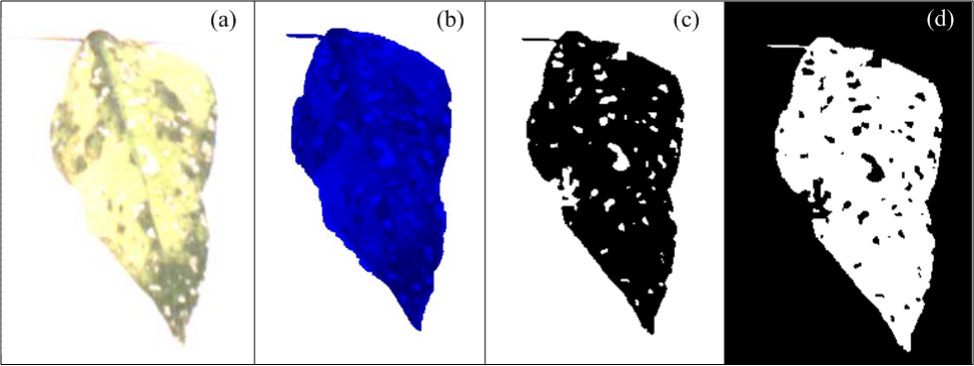
(**a**) Bean leaf with white spots. (**b**) Blue component of bean leaf with white spots. (**c**) Background and white spots segmentation. (**d**) Segmentation of leaf area without white spots.

The white-spots algorithm estimates the area occupied by white spots in leaves. They are present in the three components (Red, Green and Blue). Nonetheless, a colour-based algorithm is proposed using the blue component again because of the aforementioned reasons; Figure 9(a) shows a bean leaf in RGB format affected with white spots and Figure 9(b) shows the blue component of the original image.

First, the blue component is binarized with a predefined threshold level to isolate the leaf area from background (Figure 9(c)); this threshold level in this case was fixed but can be varied by the user according to the light conditions. After the binarization process, the number of pixels in the background is calculated by using the connectivity algorithm, later, the complement of the image is obtained and leaf area without white spots is isolated and quantified by using the connectivity algorithm (Figure 9(d)). Once we have the leaf area without white spots and the background area it is possible to obtain the area occupied by white spots *A_ws_* using Equation (11), where *A_BG_* is background area and *A_TL_* is leaf area without the white spots. The percentage of leaf area occupied by white spots *A_pws_* is estimated using Equation (12). The constant value *A_ZS_* represents the size of the image in pixels:

(11) $$A_{\mathrm{ws}}=A_{\mathrm{ZS}}-A_{\mathrm{BG}}-A_{\mathrm{TL}}$$

(12) $$A_{\mathrm{pws}}=\frac{A_{\mathrm{ws}}}{\left(A_{\mathrm{TL}}+A_{\mathrm{ws}}\right)}*100\%$$

### 3.5. Mosaic Algorithm

**Figure 10.**
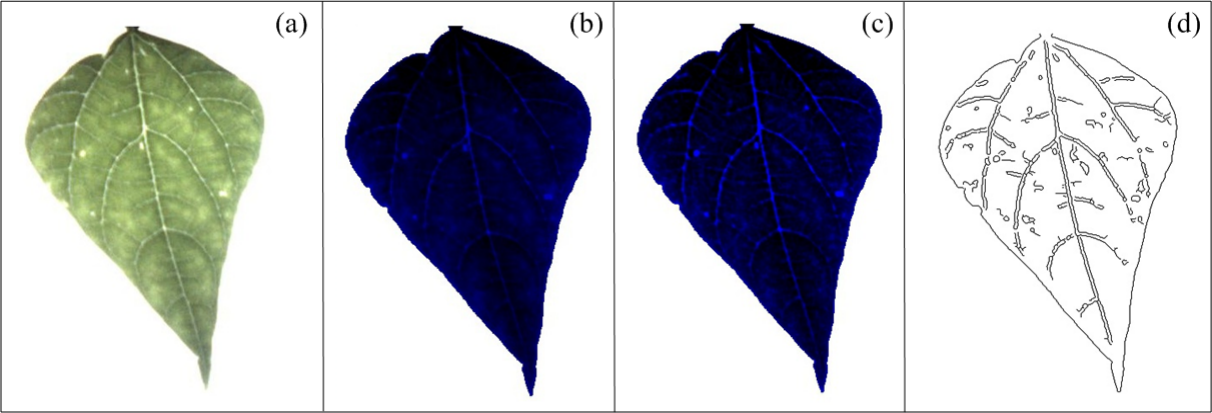
(**a**) Bean leaf in RGB format. (**b**) Blue component of bean leaf with histogram equalization. (**c**) Blue component of bean leaf with contrast enhancement after applying the top-hat and bottom-hat algorithms. (**d**) Identification of bean-leaf venation.

**Figure 11.**
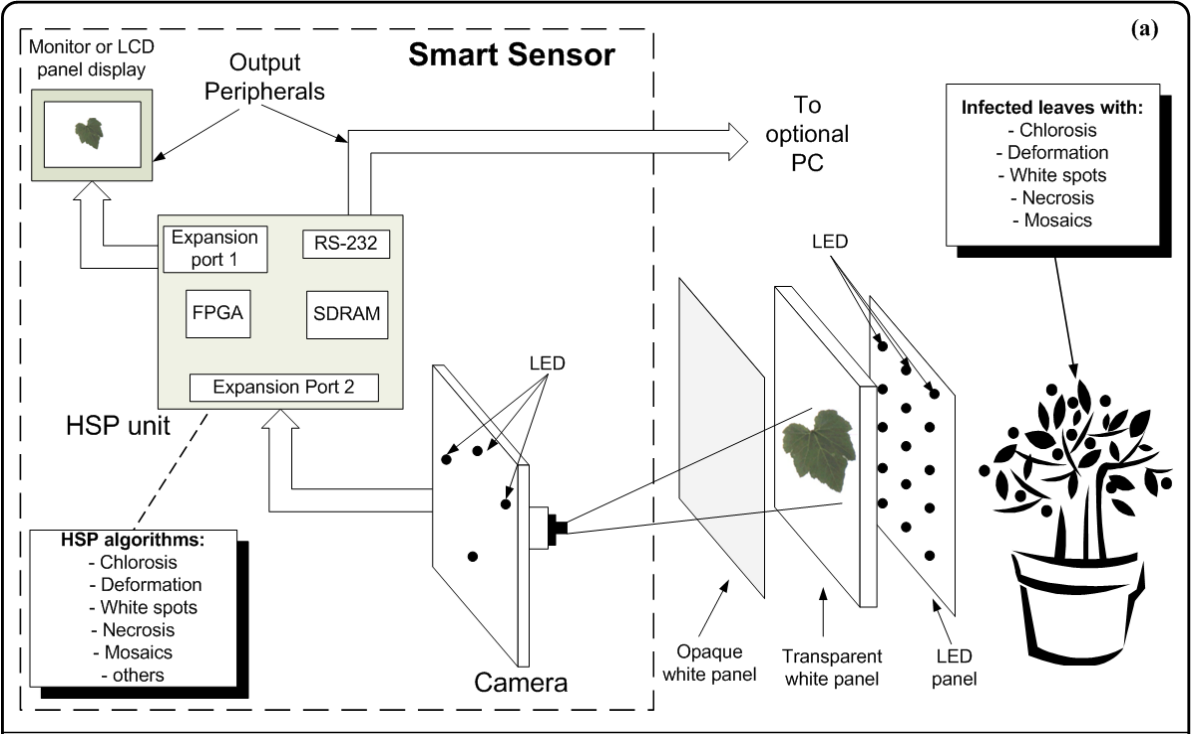

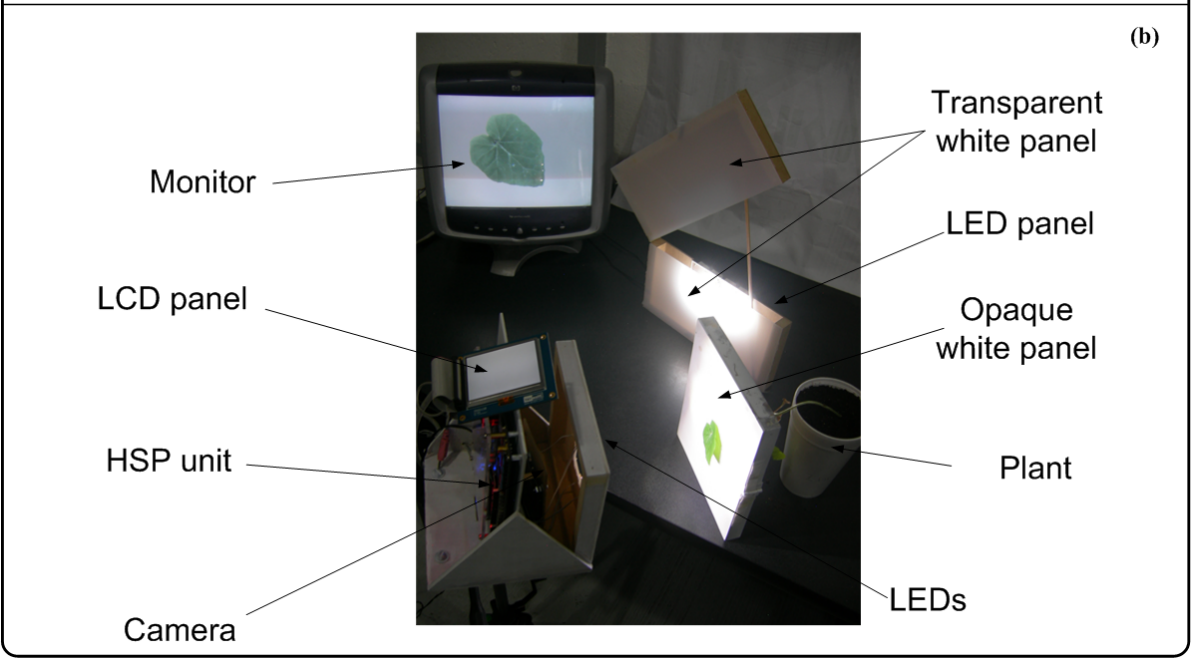
Smart-sensing methodology. (**a**) Block diagram of the proposed system. (**b**) Experimental setup.

The mosaic symptom is characterized by the presence of a high number of leaf venations in diseased leaves. This symptom is not clear when it is visualized under natural conditions, it is better detected under high-intensity and back-light conditions, as can be seen in Figure 10(a); this methodology improves the leaf venation visibility, and consequently, the mosaic symptoms can be better identified and quantified. Because of the aforementioned reasons, the blue component was used to quantify mosaic symptoms. To perform a better mosaic quantification, the plant leaf is photographed under high-intensity and back-light conditions using flash LEDs and a panel LED as can be seen in the picture of the experimental setup shown in Figure 11(b); the flash LED and LEDs of the panel LED are turned on by the user before that the plant leaf be photographed; this creates better light conditions to detect the mosaic symptoms on leaves. After the leaf has been photographed, the next step is to process the image to improve its quality in order to obtain a better differentiation between leaf venation and the leaf tissue. First, the blue component is obtained from the original image and later the gray values of blue component were distributed in the used total gray scale range (from 0 to 1,023) to enhance its contrast (Figure 10(b)), later top-hat *Thw_B_* and bottom-hat *Thb_B_* transformations are sequentially applied in already contrasted image to continue enhance its contrast according to Equations (13) and (14), and as the Figure 10(c) shows, this transformation is based on morphological opening and closing *φ_B_*, respectively, as can be seen in Equations (6) and (15) [38,39]. Finally, Canny algorithm jointly with binarization operation is applied to detect the edges of leaf venation [40] (Figure 10(d)). The quantification of the detected leaf venation is done by estimating the area covered by leaf-venation edges taking as reference the total leaf image according to Equation (16).

(13) $${\mathrm{Thw}}_{B}=A-\gamma_{B}(A)$$

(14) $${\mathrm{Thb}}_{B}=\varphi_{B}(A)-A$$

(15) $$\varphi_{B}=A•B=(A⊕B)\Theta B$$

(16) $$A_{\mathrm{mosaic}}=\frac{A_{\mathrm{map}}}{A_{\mathrm{TL}}}*100\%$$

where *B* is the structural element and *A* is the blue component image. *A_map_* is the leaf area covered with the mosaic edges, *A_TL_* is the total leaf area and *A_mosaic_* is the percentage of the area covered by mosaic edges.

## 4. Smart Sensor

The smart sensor consists of four major components: a camera as primary sensor, a Hardware Signal Processing (HSP) unit as processing element, output peripherals (LCD display panel or monitor, and an optional PC), high intensity LEDs that serve as flash lights and light sources to generate back-light conditions and three panels that are employed depending on the symptom under analysis (Figure 11). The methodology of smart-sensing cycle could be divided into three stages. In the first stage, leaf-image acquisition is done using a 1/3-inch and 1.3-megapixel CMOS active-pixel digital image sensor MT9M011 manufactured by micron [19], after this, the captured image is sent to the HSP unit, which is an Altera DE2 development kit containing an EP2C35F672C6N Cyclone II FPGA, Synchronous dynamic random-access memory (SDRAM) and peripheral ports [41].

The second stage is where the image is processed to quantify chlorotic area, deformation, white-spots area, necrotic area, and mosaic symptoms. Finally, the third stage displays the original or processed image through an LCD panel showing the results of the symptom quantification. Optionally, the image can be sent to the PC through an RS232 port to create a database in order to perform further analysis (Figure 11(a)). The three panels consist of an opaque white panel used to quantify chlorosis, leaf deformation, white spots, and necrosis; and the transparent-white and LED panel are used jointly to quantify the mosaic symptoms. The LED panel generates the high-intensity back-light that, jointly with the transparent white panel, allows that light passes through the leaf; this amplifies the visibility of the leaf venation. Figure 11(b) shows the experimental setup where all the parts can be identified in the block diagram of the proposed system. The smart sensor is mounted on a mobile mechanism that permits moving the system in order to perform *in-situ* assessments.

### HSP Unit

**Figure 12.**
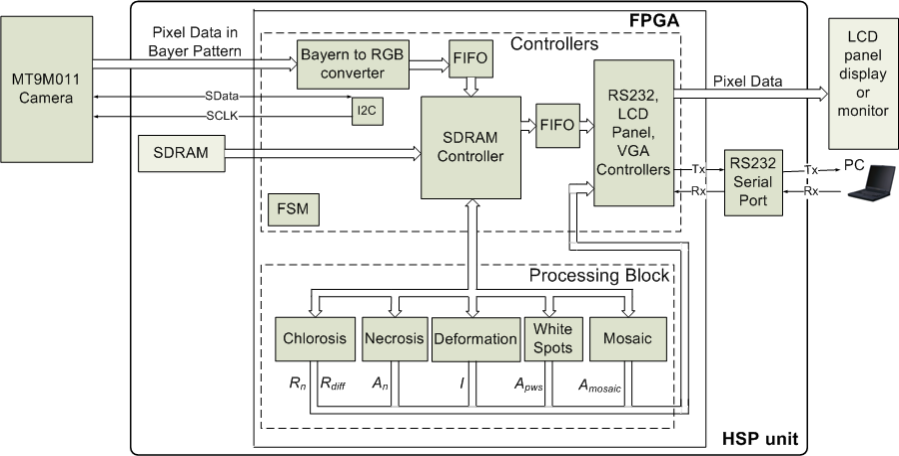
HSP-unit hardware structure.

The HSP unit is in charge of controlling the dataflow coming from the camera and performing the image processing to quantify the symptoms. The HSP unit is composed principally by four parts: FPGA, an 8M-byte SDRAM, peripheral ports (RS232) and expansion ports. The FPGA is the key component of HSP unit and it is where the image is processed and the controllers are implemented as shown in Figure 12. The controllers and processing blocks consist of Intellectual Property (IP) cores that control the devices connected to FPGA and process the image; these blocks work at different clock frequencies in order to avoid data-transfer bottlenecks between camera, SDRAM, processing blocks and peripheral ports and to accomplish the specification of the peripheral devices such as the LCD panel that operates using a 33.2-MHz clock. The SDRAM interfacing works at 100 MHz, whereas the camera interface clock is set to 25 MHz. The processing blocks, working with a 25 MHz clock, comprise five modules in charge of quantifying the symptoms by giving the values of absolute chlorosis level intensity *R_n_*; the differences between the four sections in the leaf *R_diff_*; the deformation index *I*; the percentage of necrotic leaf area *A_n_*; the leaf area percentage with white spots *A_ws_*; and the percentage of leaf area affected by mosaic symptom on the leaf *A_mosaic_*.

## 5. Results and Discussion

### 5.1. Chlorosis

**Figure 13.**
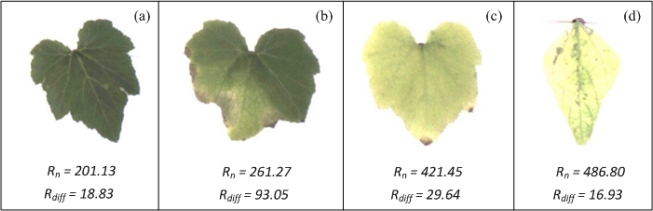
Different levels of generalized and localized chlorosis in pumpkin and pepper leaves. (**a**) Pumpkin leaf with the low-generalized and localized chlorosis. (**b**) Pumpkin leaf with high-localized chlorosis. (**c**) Pumpkin leaf with high-generalized chlorosis. (**d**) Pepper leaf with high-generalized chlorosis.

To test the chlorosis quantification methodology, pumpkin and pepper plants were used. On the one hand, pumpkin plants were put into pots for four weeks without adding any nutritional solution, provoking nutritional deficiencies in the plants that result in the development of chlorotic areas on the leaves. On the other hand, pepper plants were infected with the common viral pathogen, causing chlorosis in the leaves. Figure 13 shows leaves of pumpkin and pepper with different chlorosis levels. According to *R_n_* and *R_diff_* (values shown at the bottom of each figure) the leaf of Figure 13(a) presents the lowest values of *R_n_* and a low value of *R_diff_*, these values suggest that the leaf is almost completely covered by a green colour as can be seen in the Figure. The leaf in Figure 13(b) still has a low value of *R_n_* but has the highest value of *R_diff_*, meaning that the leaf has a high-localized chlorosis and a low-generalized chlorosis; this fact can be easily corroborated through VE done over this leaf. The leaf in Figure 13(c) show a high value of *R_n_* and a low value of *R_diff_*; indicating that the leaf has been covered almost completely by chlorotic areas (generalized chlorosis) as can be corroborated in this figure; in the same way, the leaf in Figure 13(d) show the highest value of *R_n_* and the lowest value of *R_diff_*; and these values indicate that the leaf exhibits a high level of generalized chlorosis.

### 5.2. Necrosis

**Figure 14.**
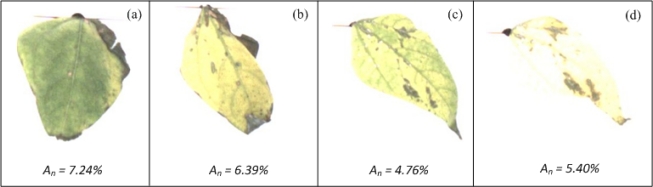
Bean and pepper leaves with different level of necrosis. (**a**) Bean leaf with necrotic and low-chlorotic areas. (**b**) Bean leaf with necrosis and high-generalized chlorosis. (**c**) and (**d**) Pepper leaves with necrosis and high-generalized chlorosis.

The necrosis algorithm was tested using bean plants infected by the common bean mosaic virus and pepper plants infected with a necrotic and bacterial disease caused by *Xanthomonas campestri*s. Bean (Figure 14(a,b)) and Pepper leaves (Figure 14(c,d)) were photographed according to the methodology depicted in Section 4. According to the values shown in Table 1, *A_n_* suggests that leaf of Figure 14(a) presents the major necrotic area and leaf of Figure 14(c) presents the minor necrotic area. The differences between each value of *A_n_* are difficult to be detected by using VE.

### 5.3. Leaf Deformation

**Figure 15.**
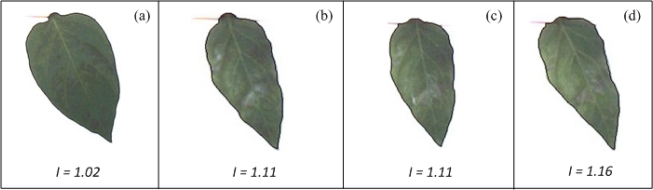
Pepper leaves with different level of deformation index. (**a**) Leaf with the lowest deformation index. (**b**) and (**c**) Leaves with intermediate deformation index. (**d**) Leaf with the highest deformation index.

The Leaf-deformation algorithm was tested using healthy and unhealthy pepper plants infected by the bacteria *Xanthomonas campestris*. Images from these plants were processed according to the methodology presented in Section 4. Figure 15 shows the shape contour of pepper leaves marked in black. According to the deformation index estimation (shown at the bottom of each figure) the leaf in Figure 15(a) has a deformation index of 1.02, which is the lowest value compared against deformation index values of leaves shown in the remaining figures (Figure 15(b–d)); similarly, the leaf in Figure 15(d) presents the highest deformation index (*I* = 1.16), while the leaf in Figure 15(b) and leaf in Figure 15(c) present an equal deformation value (*I* = 1.11), suggesting that both leaves have similar deformations. The difference between *I* value of healthy and unhealthy leaves indicate that leaves in Figure 15(b–d) are more deformed than the leaf in Figure 15(a). It can be noticed that VE can be used to identify the differences of deformation between Figure 15(a) and Figures 15(b–d); however, it cannot be used to identify the differences between Figure 15(b–d), because they have very similar forms.

### 5.4. White Spots

**Figure 16.**
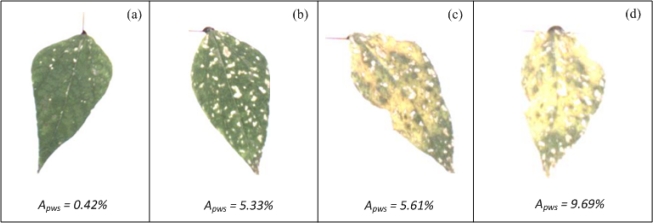
Bean leaves with different percentages of leaf area occupied by white spots. (**a**) Bean leaf with the lowest percentage of leaf area occupied by white spots. (**b**) Bean leaf with intermediate percentage of leaf area occupied by white spots. (**c**) Chlorotic-bean leaf with intermediate percentage of leaf area occupied by white spots. (**d**) Chlorotic-bean leaf with the highest percentage of leaf area occupied by white spots.

The algorithm for evaluating white-spot areas was tested using bean plants infected with the common bean mosaic virus. Figure 16 shows bean leaves with white-spots symptoms.

White-spot values *A_pws_*, shown at the bottom of each figure, suggest an incremental tendency of white spots in leaves. The leaf in Figure 16(a) is the one with the least percentage of white-spots area according to *A_pws_* and the leaf in Figure 16(d) is the leaf with the highest percentage of white-spots area. Leaves shown in Figure 16(b,c) have intermediate values. The differences between Figure 16(a) and Figure 16 (b–d) are easily identified through VE; however, VE cannot be used as reliable tool to indicate the differences between the leaves in Figure 16(b–d).

### 5.5. Mosaic

**Figure 17.**
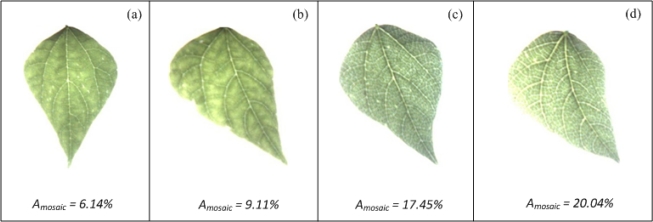
Bean leaves with different percentages of area covered by mosaic symptom. (**a**) and (**b**) Healthy bean leaves with the lowest percentage of area covered by mosaic symptom. (**c**) and (**d**) Unhealthy bean leaves with the highest percentages of area covered by mosaic symptom.

The Mosaic-algorithm was tested using bean plants infected with the common bean mosaic virus that causes the mosaic veinclearing symptom. Figure 17 shows leaves with and without mosaic symptoms; *A_mosaic_* values shown in Figure 17 suggest that the leaves of Figure 17(a,b), which are the healthy leaves, have a lesser percentage of area covered by mosaic symptoms than the unhealthy leaves of the Figure 17(c,d). It is important to mention that, the differences of mosaic symptoms between Figure 17(a,b) and between Figure 17(c,d) cannot be easily identified through VE.

### 5.6. FPGA Implementation

The processing blocks implemented in the FPGA of the HSP unit (Figure 12) use a 25-MHz clock; the processing times employed by each block to accomplish their corresponding function are shown in Table 1.

The implementation makes use of 7,403 logic elements, 34 9-bit multipliers, and 235,616 memory bits from the FPGA, corresponding to 22.29%, 48.57%, and 48.70% usage, respectively. It can be noticed that all image processing algorithms are below 1 s in execution time, which guarantees the *in situ* and online processing capabilities of the implemented system. Besides, the FPGA has more than 50% of resources available for incorporating further processing algorithms.

## 6. Conclusions

The present work describes a smart sensor capable of providing precise, reliable and robust quantification of common symptoms presented in leaves of diseased and malnourished plants; these symptoms are: chlorosis, necrosis, deformation, white spots and mosaics. The smart sensor employs a 1/3-inch megapixel CMOS active-pixel digital image sensor (camera) that acts as primary sensor and a Altera-DE2 development card containing an EP2C35F672C6N-Cyclone II FPGA that serves as processing element because it contains enough embedded resources to satisfy the high-computational demand of the proposed algorithms. Novel colour-based methodologies and FPGA characteristics are exploited by the smart sensor to give quantitative, *in-situ*, precise, real-time, and non-destructive symptom assessments. The functionality of the smart sensor was successfully tested by analyzing the aforementioned five common symptoms present in leaves of diseased and malnourished plants. The comparative analysis of the results shows that the quantitative values of Figures 13–17 prove the reliability and precision of the smart sensor in giving quantitative assessments of symptoms that are present in leaves of plants; in consequence, the proposed system could be employed in future works as a practical and powerful tool to give, through plant-leaf symptom quantification, a precise and reliable quantitative description of syndrome development of diseased plants or to determine quantitatively the nutritional deficiencies of plants. This work can also serve as a tool to know the resistance of specific plant varieties to certain pathogens and by consequence help develop new more resistant varieties. For further development, the FPGA reconfigurability capabilities allow the incorporation of different algorithms able to estimate other symptoms not considered in this work.

## Figures and Tables

**Table 1.** Processing times of the blocks implemented in the FPGA.

**Processing block**	**Processing time (ms)**
*Chlorosis*	123.398
*Necrosis*	12.289
*Deformation*	48.490
*White spots*	264.192
*Mosaic*	354.080
